# Increased Rate of Fracture Injuries Associated With Alternative Modes of Transportation During COVID-19

**DOI:** 10.5435/JAAOSGlobal-D-22-00147

**Published:** 2022-09-27

**Authors:** Sam H. Jiang, Max Davison-Kerwood, Mark H. Gonzalez

**Affiliations:** From the University of Illinois College of Medicine at Chicago (Mr.Jiang, Mr.Davison-Kerwood, and Dr. Gonzalez), and the Department of Orthopaedic Surgery, University of Illinois at Chicago, Chicago, IL (Dr. Gonzalez).

## Abstract

COVID-19 disrupted public transit and led to increased reliance on alternative modes of transportation (AMTs) internationally. This study hypothesizes that public interest and fracture injuries associated with AMTs increased during COVID-19 in the United States.

**Methods:**

Monthly Google search probabilities and the number of fracture injuries associated with bicycles, scooters, skateboards/longboards, rollerblades, electric bicycles, and electric micromobility vehicles were collected from January 2017 to December 2021. Wilcoxon signed-rank tests were used to assess differences in search probabilities and fracture injuries between 2021, 2020, and 2019. Linear regression was used to study the relationship between search probabilities and number of fracture injuries.

**Results:**

For bicycles, skateboards/longboards, electric bicycles, and electric micromobility vehicles, search probabilities and fracture injuries were higher in 2021 and 2020 compared with 2019, except for bicycle fractures in 2021 (*P* < 0.05). For every AMT, except roller skates, search probability had an explanatory effect on fracture injuries (*P* < 0.001).

**Conclusion:**

Online interest in AMTs and associated fracture injuries increased during the COVID-19 pandemic. Excess fractures seem to be stabilizing as of December 2021, but online search volumes may be used to inform the allocation of orthopaedic trauma resources during future surges in COVID-19 and other epidemics.

In early 2020, the SARS-CoV-2 virus rapidly spread across the world at an unprecedented rate, giving countries little to no time to enact preventive public health measures. Seeking to manage the transmission of the novel COVID-19 pandemic, governments raced to enact emergency social and legal regulations such as social distancing mandates, limits to public transit capacity, and restrictions on public gatherings. As a result, several cities across Europe and Asia saw decreases in public transit ridership because of the public fear of infection.^1-3^ At the same time, those cities witnessed a shift in transportation reliance toward alternative modes of transportation (AMTs), such as bicycles and scooters, which allowed individuals to adhere to social distancing and other quarantine guidelines more easily.^1,3^ Even before COVID-19, however, the use of AMTs had been on the rise because nations and individuals sought to reduce their carbon footprint to combat the impending climate change crisis. Because of this trend, AMT use has been linked to a multitude of injuries from minor ones such as skin abrasions, lacerations, and overuse injuries to major ones such as bone fractures, traumatic brain injury, and even death.^4,5,6,7^ Considering this preexisting multifactorial rise in the use of AMTs, coupled with the shift toward conservative management for fracture injuries during COVID-19 to limit disease transmission, a thorough understanding of the trends and driving forces behind intrapandemic fracture injury rates will help direct care for the remainder of the COVID-19 pandemic and future pandemics alike.^8-10^

This study seeks to evaluate whether the number of AMT-associated fracture injuries increased during the COVID-19 pandemic in the United States and assess its association with online search interest in AMTs. To that end, this study makes use of a novel method to approximate individual usage: online search probabilities. The Google Extended Trends Application Programming Interface (API) for Health (GETH), a closed access version of Google Trends designed for health research, is a tool that measures public interest in a specific subject. GETH calculates the probability that a given term is searched on Google in a specified time and area, enabling researchers to analyze how public interest changes over time and during major events such as COVID-19. The use of GETH in health research has increased 20-fold between 2008 and 2018, and its effectiveness in measuring public interest has been demonstrated in studies focused on elective surgeries, influenza outbreaks, depression, HIV/AIDS, and much more.^11,12,13,14,15^ Leveraging the epidemiological effect of GETH, this study hypothesizes that in the United States, both public interest in AMTs and AMT-associated fracture injuries increased during COVID-19.

## Methods

**Figure 1:**
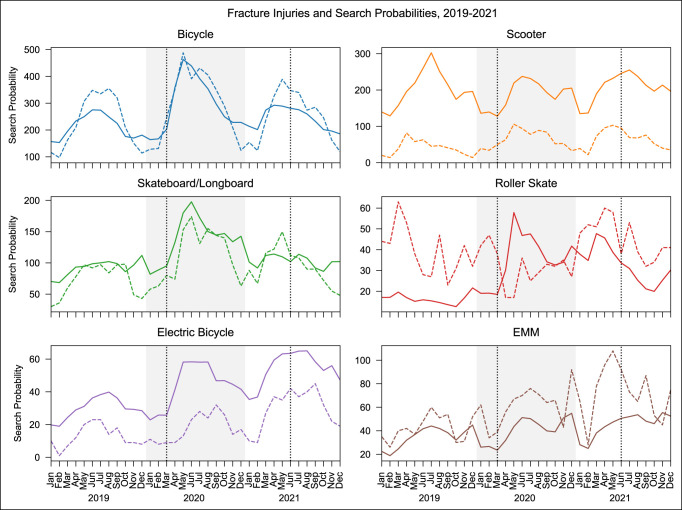
Graphs showing the number of fracture injuries and search probabilities between January 1, 2019, and December 31, 2021, for bicycles, scooters, skateboards and longboards, roller skates, electric bicycles, and electric scooters, longboards, and skateboards. In each plot, the first dotted vertical line corresponds to the beginning of COVID-19 in the United States (March 2020) and the second corresponds to the beginning of the Delta variant surge (June 2021). Probabilities are reported as 0.2 × *P* × 10^8^ for bicycles and *P* × 10^8^ for all others.

Using the GETH Python API, search probabilities were collected for bicycles, scooters, skateboards/longboards, rollerblades, electric bicycles, and electric micromobility vehicles (EMMs), which include powered scooters, skateboards, and longboards. The composite search queries used to collect search probabilities for each AMT are provided in Appendix Table 1, http://links.lww.com/JG9/A241. Data were collected across the entire United States for every month between January 1, 2017, and December 31, 2021, resulting in 60 data points for each AMT (Figure 1). Fracture-related injuries for each AMT were then collected from the annual National Electronic Injury Surveillance System (NEISS) reports. Diagnosis code 57 was used to identify fracture injuries, and then, product codes were used to identify fractures related to each AMT. The specific product codes and labels used are presented in Appendix Table 2, http://links.lww.com/JG9/A242. Fracture data were collected for each month in the same time window, resulting in 60 data points corresponding to the GETH data.

**Figure 2:**
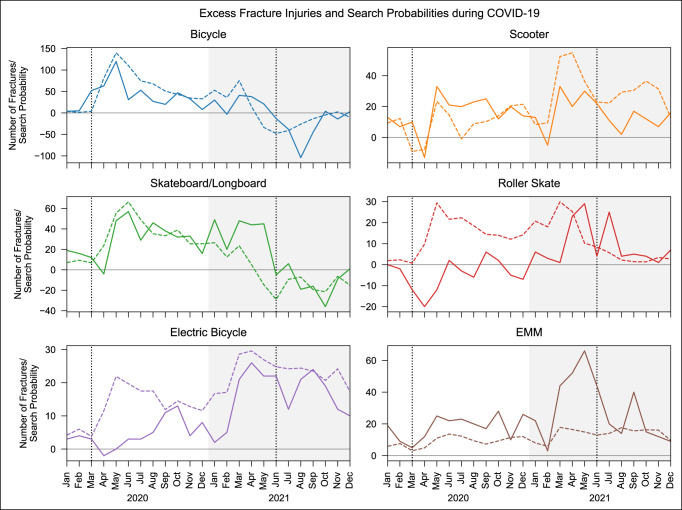
Graphs showing excess number of fracture injuries and search probabilities in 2020 and 2021 compared with 2019. In each plot, the first dotted vertical line corresponds to the beginning of COVID-19 in the United States (March 2020) and the second corresponds to the beginning of the Delta variant surge (June 2021). Probabilities are reported as 0.2 × *P* × 10^8^ for bicycles and *P* × 10^8^ for all others.

The mean of monthly fracture injuries and search probabilities for each AMT were determined as a historical average. Then, the difference between the 2020 and 2021 values and the historical average for each AMT was calculated and plotted to visualize “excess” fracture injuries and online search interest throughout the pandemic (Figure 2).

For each AMT, Wilcoxon signed-rank tests were then conducted between the monthly numbers of fracture injuries for each AMT in 2020 versus 2019, 2021 versus 2019, and 2021 versus 2020, for a total of 18 comparisons. Another 18 analogous Wilcoxon tests were then conducted with monthly search probabilities. An ordinary least squares (OLS) regression with a random constant was used for each AMT with search probability as the independent variable and the number of AMT-related fracture injuries as the dependent variable, for a total of six OLS analyses. In addition, the monthly excess number of fracture injuries and search probabilities were calculated by subtracting the monthly values in 2020 and 2021 from the monthly averages in 2017, 2018, and 2019.

As a corollary to the hypothesis, to assess the initial transportation-related public response to COVID-19, a secondary analysis was conducted to assess the relationship between city-wide public transit ridership and online interest in AMTs in 2020. Using the American Community Survey by the United States Census Bureau, the percent ridership of 25 US cities with available designated market codes (which are used to localize GETH data) was collected. An OLS regression with a random constant was used with percent ridership as the independent variable and change in interest for each AMT between 2020 and 2019 as the dependent variable. The cities and Designated Market Area codes used are given in Appendix Table 3, http://links.lww.com/JG9/A243.

## Results

The Wilcoxon results showed that for bicycles, scooters, skateboards/longboards, electric bicycles, and EMMs, both search probabilities and number of related fracture injuries were markedly higher in both 2021 and 2020 compared with 2019, except for bicycle fractures in 2021 compared with 2019. Between 2021 and 2020, fracture injuries increased for EMMs and roller skates and decreased for bicycles while search interest increased for scooters, electric bicycles, and EMMs, and decreased for skateboards/longboards (Tables 1 and 2).

**Table 1:** Number of Fracture Injuries Associated With Each Alternative Mode of Transportation in 2020 Versus Previous Years

AMT	Year 1 Mean	Year 2 Mean	Cohen D	*P*
2021 vs. 2020				
Bicycles	250.08	295.33	−0.41	0.005^a^
Scooters	64	64.58	−0.02	0.58
Skateboards/longboards	93.17	111	−0.48	0.18
Roller skates	45.58	31.5	1.53	<0.001^a^
Electric bicycles	29.58	17.83	1.12	0.001^a^
EMMs	72.08	61.67	0.52	0.3
2021 vs. 2019				
Bicycles	250.08	227	0.24	0.09
Scooters	64	40	1.01	<0.001^a^
Skateboards/longboards	93.17	71.75	0.76	0.03^a^
Roller skates	45.58	39.25	0.6	0.08
Electric bicycles	29.58	12.83	1.69	0.003^a^
EMMs	72.08	42.17	1.68	<0.001^a^
2020 vs. 2019				
Bicycles	295.33	227	0.6	<0.001^a^
Scooters	64.58	40	1.07	0.003^a^
Skateboards/longboards	111	71.75	1.11	<0.001^a^
Roller skates	31.5	39.25	−0.74	0.15
Electric bicycles	17.83	12.83	0.65	0.04^a^
EMMs	61.67	42.17	1.4	<0.001^a^

AMT = alternative mode of transportation, EMM = electric micromobility vehicle

a Significant.

Each row represents the Wilcoxon signed-rank test results for the number of fracture injuries associated with each AMT in 2020 versus the year denoted in the subheading mentioned earlier.

**Table 2:** Search Probability of Each Alternative Mode of Transportation in 2020 Versus Previous Years

AMT	Year 1 Mean	Year 2 Mean	Cohen D	*P*
2021 vs. 2020				
Bicycles	12,094.38	14,761.74	−0.68	0.06
Scooters	2,061.55	1,869.56	0.5	0.009^a^
Skateboards/longboards	1,028.84	1,388.76	−1.37	0.009^a^
Roller skates	326.06	352.55	−0.24	0.79
Electric bicycles	544.31	440.19	0.88	<0.001^a^
EMMs	450.43	402.73	0.46	0.01^a^
2021 vs. 2019				
Bicycles	12,094.38	10,574.5	0.71	<0.001^a^
Scooters	2,061.55	2,028.18	0.07	0.52
Skateboards/longboards	1,028.84	917.92	0.99	0.03^a^
Roller skates	326.06	163.69	2.48	<0.001^a^
Electric bicycles	544.31	300.87	2.79	<0.001^a^
EMMs	450.43	346.98	1.11	<0.001^a^
2020 vs. 2019				
Bicycles	14,761.74	10,574.5	1.05	<0.001^a^
Scooters	1,869.56	2,028.18	−0.35	0.09
Skateboards/longboards	1,388.76	917.92	1.74	<0.001^a^
Roller skates	352.55	163.69	2.1	<0.001^a^
Electric bicycles	440.19	300.87	1.32	<0.001^a^
EMMs	402.73	346.98	0.56	0.002^a^

AMT = alternative mode of transportation, CI = confidence interval, EMM = electric micromobility vehicle

a Significant.

Each row represents the Wilcoxon signed-rank test results for the Google search probability of each AMT in 2020 versus the year denoted in the subheading mentioned earlier.

**Figure 3:**
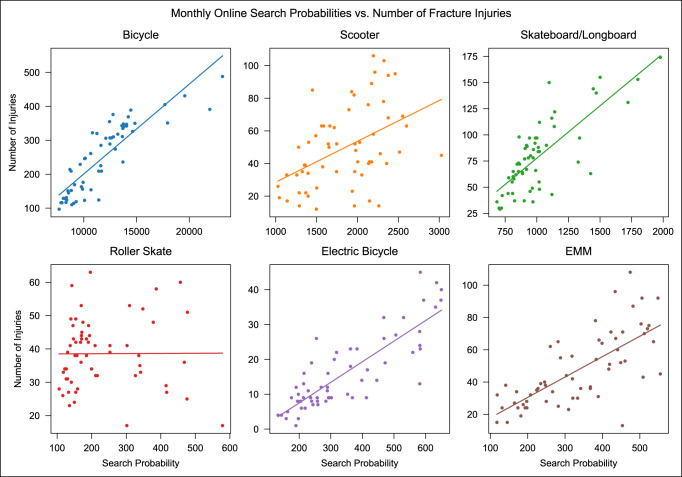
Graphs showing monthly search probabilities versus the number of fracture injuries with best-fit lines for each mode of transportation. Probabilities are reported as *P* × 10^7^.

The linear regression results revealed that for every AMT, except roller skates, search probability had a notable effect on the number of fracture injuries. Bicycles, scooters, skateboards/longboards, electric bicycles, and EMMs had *P* < 0.001, with R^2^ values of 0.75, 0.21, 0.6, 0.7, and 0.51, respectively (Table 3 and Figure 3). The corollary analysis revealed that percent ridership in cities had an explanatory effect on the increased interest in 2020 for bicycles (R^2^ = 0.24, *P* = 0.01), but did not have a significant effect on the other AMTs (Table 4).

**Table 3:** Linear Regression Results Between Search Probability and Number of Associated Fracture Injuries of Each Alternative Mode of Transportation

AMT	R2	R	Coefficient	Lower CI	Upper CI	*P*
Bicycles	0.75	0.87	0.03	0.02	0.03	<0.001^a^
Scooters	0.21	0.46	0.03	0.01	0.04	<0.001^a^
Skateboards/longboards	0.6	0.78	0.1	0.08	0.12	<0.001^a^
Roller skates	<0.001	0.005	<0.001	<0.001	0.02	0.97
Electric bicycles	0.7	0.84	0.06	0.05	0.07	<0.001^a^
Powered devices	0.51	0.72	0.13	0.09	0.16	<0.001^a^

AMT = alternative mode of transportation, EMM = electric micromobility vehicle

a Significant.

**Table 4:** Linear Regression Results Between City-wide Percent Ridership and Increase in Search Interest in 2020 for Each Alternative Mode of Transportation

AMT	R2	R	Coefficient	2.5% CI	97.5% CI	*P*
Bicycles	0.24	0.49	38.17	9.13	67.22	0.01^a^
Scooters	0.004	0.06	1.64	<0.001	13.21	0.77
Skateboards and longboards	0.009	0.1	<0.001	<0.001	3.24	0.65
Rollerblades	0.05	0.22	0.72	<0.001	2.14	0.3
Electric bicycles	0.09	0.3	1.46	<0.001	3.44	0.14
EMMs	0.002	0.04	0.26	<0.001	2.86	0.84

AMT = alternative mode of transportation,CI = confidence interval, EMM = electric micromobility vehicle

a Significant.

## Discussion

The goal of this study was to determine whether the number of AMT-associated fracture injuries increased in 2020 and 2021 after the novel COVID-19 pandemic in the United States and to assess the association of online search interest in AMTs with those fracture injuries, as measured through Google search probabilities. In addition, this study sought to discern whether areas in the United States that are more reliant on public transportation were more acutely affected in the first year of the pandemic. This study showed that as a whole, both the number of AMT-associated fracture injuries and online search interest increased in 2020 and 2021 compared with 2019 (Figure 1), with the latter having a significant explanatory effect on the former (Figure 3). Furthermore, the areas most dependent on public transport were more acutely affected for bicycle interest, suggesting that bicycles may be viewed favorably as a practical and affordable alternative to public transportation, whereas the remaining AMTs are not (Table 4). These findings highlight transportation-related injuries as a previously unidentified area of health care that has been negatively affected by the COVID-19 pandemic in the United States.

Interestingly, the spike in cases due to the Delta variant in June 2021 seems to have had a paradoxical effect. Contrasted with the across-the-board increases in both search interest and fracture injuries at the start of COVID-19, the Delta surge triggered a decrease in search interest for roller skates and electric bicycles and, more importantly, a decrease in fracture injuries for bicycles, scooters, electric bicycles, and EMMs (Figure 2). One possible explanation for this paradoxical finding is that compared with the beginning of the pandemic, people in June 2021 were much more aware of the dangers of COVID-19 and were more likely to self-quarantine and avoid outdoor transit as the number of cases and media coverage of the Delta variant increased. Another explanation is many people returned to in-person jobs by June 2021, so the onset of the Delta surge resulted in another transition to work-from-home employment, resulting in a decline in AMT-based commuting and subsequent injuries.

### Alternative Modes of Transportations for Transportation Versus Recreation

When looking at Figure 1, two annual patterns of interest probability emerge. For bicycles and electric bicycles, the largest annual spike occurs during the summer months, whereas for skateboards/longboards and EMMs, the largest spike occurs around November and December. Scooters display a hybrid pattern, with a primary spike in summer and a smaller but notable spike in December. A study by Gudeman et al found similar results that may explain this phenomenon. Their results showed that emergency department visits for scooter-related injuries peaked in spring and summer for all modalities, except hoverboards, which peaked in December and are typically given as a holiday gift.^16^ These findings suggest that AMTs can be divided into two primary categories: those used mainly for transportation, which display peak interest during the warmer summer months when they are more viable, and those used mainly as novelties or recreation, which display peak interest during the gift-giving holiday season. This delineation between transportation and recreation is supported by previous studies.^17-19^ The transportation-versus-recreation divide is a potential explanation for why the increased interest in bicycles and electric bicycles during COVID-19 correlated with public transit ridership. Both are primarily used for transportation, but other AMTs were not.

### Increase in Bicycle Usage During COVID-19

An increase in bicycle usage during COVID-19 has been observed in previous studies. Schaefer et al surveyed 4,000 participants in Hannover, Germany, in June 2020 about their bicycle-use habits before and during the pandemic. They found that train and bus use was replaced by bike and car use, with an emphasis on train use being replaced by bike use. In addition, they found that fear of infection was a driving factor for reduced public transit usage and that bike use was dependent on the eco-consciousness rather than the socioeconomic status of users.^3^

In another study, Nikitas et al^1^ conducted a thorough review of 88 case studies of procycling initiatives that have been implemented worldwide in response to the COVID-19 pandemic. In this review, they specified reduced public transit ridership, as well as narrow streets and insufficient prowalking and procycling infrastructure in cities worldwide, as key motivators for these procycling initiatives. In the United States, they identified several trends across the country that support the notion of increased cycling during COVID-19. In New York City (NYC), cycling activity on East River bridges increased by 50% in 2020 compared with 2019, as reported by the NYC Department of Transportation.^1^ In Philadelphia, cycling increased by 151% on city trails, leading city officials to implement the Vision Zero initiative to reduce cycling accidents.^20^ In addition, although ride-sharing frequency decreased, the average ride duration increased during the pandemic,^21^ and rideshare services such as Divvy in Chicago rebounded faster at the beginning of the pandemic than public transportation, private automobiles, and other forms of transportation.^22^

The public in major cities was more sensitive to the effects of COVID-19 on transportation habits as people shifted to bicycling and scootering as practical alternatives. As such, public outreach programs to increase driver awareness and rider safety would be most impactful in areas such as New York City and Washington, DC, to reduce the fracture burden and emergency department censuses in these areas.

### Rise of Electric Bicycles and Electric Micromobility Vehicles in Recent Years

One phenomenon that may help explain some of the results of this study is the rise in both public interest and usage of electric bicycles, scooters, and other EMMs over the past 5 years. Although vehicles have existed for decades, a shift toward the mainstream occurred in the fall of 2017, when US-based company Bird introduced their affordable, convenient, and environment-friendly scooters to the market.^23^ Since then, e-scooters have become a billion-dollar industry in the United States, and people across the country are embracing e-scooters, either through private ownership or ride-sharing services. This trend is also seen in cities around the world, with one study by Haworth et al finding that private ownership of electric scooters tripled in the 8 months between February and October 2019.^24^ The rapid and sweeping adoption of EMMs in cities across the world suggests that they are here to stay for the foreseeable future.

Despite their popularity, electric bicycles, scooters, and other EMMs are relatively new modes of transportation. As such, both government safety regulations and public awareness about their associated dangers are lacking. In addition, there is no system in place to provide helmets and other protective equipment to rideshare service users, despite their increased popularity.^7^ Because of these gaps in safety measures, EMMs have been linked to increased risk of musculoskeletal injuries in several studies.^4,5,25,26,27,28,29^ At the same time, studies have identified other, less common risks such as burn injuries from exploding batteries and potential environmental harm due to inappropriate battery disposal in water sources.^30-32^ Given the already increasing popularity of EMMs, the fact that interest and injury rates increased even more during 2020 and 2021 is alarming. The COVID-19 pandemic intensifies the need for public awareness about the dangers of EMMs and for safety measures to mitigate these dangers.

### Increased Focus on Conservative Management

Medical management of fracture care was acutely affected during the COVID-19 pandemic secondary to government regulations, personnel and resource shortages, and public fear of viral transmission in the hospital setting.^33^ Because of these factors, providers adopted higher thresholds for surgical management of fractures. Instead of surgery, older, conservative methodologies for the medical treatment of fractures were revisited, focusing on reduction and maintenance of reduction until resolution took place. The increased rates of AMT-associated fractures reported in this study, coupled with this change in fracture care, offer a unique look into both the etiology and management of fracture injuries during a worldwide pandemic. Lessons from this pandemic teach us that conservative fracture management skills must not be lost in favor of surgical intervention in the modern world because the latter may not always be a viable option. Additional research is warranted on the outcomes of patients who received conservative care during the pandemic to understand the long-term effect of the pandemic on AMT-associated and unrelated fracture injury patients.

### Limitations

This study must be viewed within the scope of its limitations. This study uses Google search probabilities as a proxy for large-scale public interest in the use of AMTs as a mode of transportation. Because Google is unable to assess user intent, it obligately captures nonusage-related interest in AMTs, subsequently inflating the data. This study uses Google search probabilities in a relative manner, which circumvents this limitation to some extent. This study does assume that the inflation in search probability is proportional across all queries; however, there is no present way to verify this assumption. NEISS relies on a network of approximately 100 hospitals of the 5,000+ hospitals in the United States to collect a representative sample of data. Although NEISS represents a stratified probability sample of all US hospitals that have at least six beds and provide 24-hour emergency department services,^34^ a notable portion of those hospitals are major medical centers in large metropolitan areas such as Los Angeles, Chicago, and Washington, DC, with a large concentration of hospitals in the Northeast. As such, the injury data may be skewed toward urban populations in those areas while underrepresenting rural populations in the Midwest, Central, and Southern United States. Furthermore, NEISS reports all injuries associated with specific consumer products, regardless of the cause of injury, so the number of AMT-caused injuries may be lower than the numbers reported in this study.

## Conclusion

At the onset of the COVID-19 pandemic in the United States, public institutions, private organizations, and individuals alike changed their behaviors to manage the spread of the virus. As public transit systems shut down and unemployment rates increased, people sought safer alternatives for both transportation and recreation, resulting in an increase in online search interest in AMTs in 2020 and 2021. As an unfortunate but not unsurprising result of this trend, fracture injuries associated with AMTs also rose during this period. Luckily, as of December 2021, it would seem that both search interest and the number of fracture injuries for nonpowered devices are close to or below prepandemic levels, although electric bicycle and EMM levels remain high (Figure 2). In addition, the paradoxical decrease in injury dates at the onset of the Delta surge suggests that the public may adopt a more cautious attitude toward AMT-based travel during future surges. Although not conclusive, these findings suggest that the pandemic-induced “fracture burden” in 2021 is greatly reduced compared with 2020, and conditions may continue to improve in 2022 as the pandemic continues to resolve in the United States.

Although the findings of this study suggest that the worst has passed, several important lessons can be learned for the future for orthopaedic trauma services and public health agencies alike. After events that disrupt public transit systems, increased demand for fracture care may be seen nationally, especially in cities. In response, campaigns to increase awareness of the risks and dangers of AMT use, increased public access to helmets and other safety equipment, and support for procycling city regulations may help mitigate fracture-related healthcare burden. This effect can be exacerbated during the summer months or holiday season as demand for AMTs for both transportation and recreation increases or as the popularity of EMM devices and electric bike-sharing services continues to increase. Clinicians should be aware of these trends and be prepared to adapt to both an increased patient volume and increased barriers to care (such as social distancing) during future COVID-19 waves, other pandemics, or similar events. Although vaccination rates improve, COVID-19 cases decrease, and restrictions are slowly lifted as the pandemic continues into its third year, these trends should continue to be monitored in 2022 to gain a full understanding of the effect of the pandemic on transportation trends in the United States.
